# Dental anomalies in an Albanian orthodontic sample: a retrospective study

**DOI:** 10.1186/s12903-023-02711-x

**Published:** 2023-01-28

**Authors:** Franceska Vinjolli, Megi Zeqaj, Edi Dragusha, Arianna Malara, Carlotta Danesi, Giuseppina Laganà

**Affiliations:** 1 Department of Surgical Science, Catholic University Our Lady of Good Counsel, Tirana, 1001 Albania; 2 Tirana, Albania; 3 Department of Economics and Business Sciences, Catholic University Our Lady of Good Counsel, Tirana, 1001 Albania; 4 Department of Systems Medicine, University of Rome “Tor Vergata”, Viale Oxford 81, 00133 Rome, Italy; 5 UniCamillus - Saint Camillus International University of Health Sciences, Rome, Italy

**Keywords:** Dental anomalies, Panoramic radiograph, Hypodontia, Tooth impaction

## Abstract

**Background:**

To evaluate the prevalence and the distribution of dental anomalies in an Albanian orthodontic sample.

**Methods:**

For this retrospective study, a sample of panoramic radiographs (PR) of n. 779 (456 F and 323 M, mean age of 15.1 ± 5.5 years) Albanian subjects with no genetic syndromes or craniofacial malformations (e.g., cleft lip/palate), history of extraction, trauma or previous orthodontic treatment drawn from the archives of the University Dental Clinic Our Lady of Good Counsel, was examined. The inclusion criteria were: subjects from 8 to 30 years of age, good quality of PRs in order to allow the assessment of crown and root development. For different dental anomalies, both the prevalence and the association were evaluated by using Chi-square test (*p* < 0.05).

**Results:**

24.4% of the sample had at least one dental anomaly and 4.6% had more than one. The following dental anomalies were most prevalent: hypodontia (9.8%), dental impaction (7.6%), and ectopic eruption (5.3%). No statistically significant correlation was found between males and females (*p* > 0.05).

**Conclusions:**

The most common anomalies in this group are found to be those of number and position, and only radiological investigations can reveal either of these anomalies. Early diagnosis of dental anomalies can contribute to prevent their complications and to establish the most suitable therapy to achieve a functional occlusion.

## Backgrounds

Dental anomalies (DA) are congenital defects that can appear alone or as part of a syndrome. [1]. Dental anomalies can be classified by size, shape, number, structure and by eruption or exfoliation alteration [2]. Complex interactions between genetic, epigenetic and environmental factors can affect the normal process of dental development [3]. This can lead to functional, occlusal and aesthetic problems due also to alterations to the process of dental eruption, so an early intervention is crucial under certain conditions [4]. Several epidemiological studies have been conducted in different populations showing a different prevalence ranged between 5.5 and 74.7%. This wide range can be explained by the different ethnicity, study methodology and diagnostic criteria [5–9]. In several studies, dental anomalies are analysed by using only the panoramic data. Pallikaraki [6] reported a prevalence of dental anomalies equal to 18.7% in Greek orthodontic population, where males were affected more frequently than females and the most common dental anomaly seems to be the oligodontia. Laganà [7] studied a no-orthodontic Italian population and the results have shown that the overall prevalence of dental anomalies was 20.9%, with a male/female ratio of 1:1. The most frequent anomaly was the canine displacement (7.5%) followed by hypodontia (7.1%). Other authors included clinical and radiographic information for the diagnosis like Baron [8], who reported that 45.7% of the French study population have presented at least one anomaly with no statistically significant correlation between genders. Fekonja [9] proved, in the Slovenian population, that 16.7% of the subjects had dental developmental anomalies where the most frequent was hypodontia. Meanwhile, there are no data regarding the Albanian population.

Several studies evidenced that the absence of the maxillary lateral incisors may be considered a diagnostic marker for the displacement of the maxillary canine [10, 11]. Therefore, early detection of dental development anomalies can contribute to establish different treatment modalities to achieve a functional dentition in the future and to prevent the complications of dental anomalies development.

The aim of this study was to evaluate the prevalence and the distribution of dental anomalies in an Albanian orthodontic sample.

## Methods

This study followed the principles laid down by the World Medical Assembly in the Declaration of Helsinki 2008 on medical protocols and ethics and it was approved by the Ethical Committee of the Catholic University Our Lady of Good Counsel (protocol number: 442). Written consent was obtained from all subjects included in the study.

An initial sample of digital and analogue panoramic radiographs (PR) of 1300 Albanian subjects was randomly selected drawn from the archives of the University Dental Clinic Our Lady of Good Counsel. The inclusion criteria were: age between 8 and 30 years, Albanian subjects, with no genetic syndromes or craniofacial malformations (e.g., cleft lip/palate), endocrine imbalances, metabolic or hereditary disorders, history of extraction, trauma or previous orthodontic. The minimum limit of age (≥ 8 years old) was chosen to view the gems of all the dental elements, except the third molars which were not taken into consideration for the present study.

All PRs were collected from January 2020 to December 2021. From the initial sample of 1300 PRs, 779 PRs of subjects were selected.

The studied sample was divided into two subgroups according to the participants’ age (Table 1):

- Group 1 (G1): 138 subjects aged between 8 and 10 years old (74 F, 64 M, mean age of 8.8 ± 0.6 years)
- Group 2 (G2): 641 subjects aged between 11 and 30 years old (382 F, 259 M, mean age of 16.4 ± 5.1 years).
- The panoramic radiograph images were evaluated independently by one single operator (M.Z.) on the computer monitor for digital radiographs and on a diaphanoscopic in a room with no natural light for analogue radiographs. To estimate the reproducibility of diagnosis a sample of 100 randomly selected radiographs were examined once again separately by the same operator. A paired *t-test* was used to compare the two measurements (systematic error).
- Dental anomalies were ranking as follow:
- Tooth number alterations: Hypodontia, excluding third molars, oligodontia, anadontia and supernumerary teeth
- Tooth size alterations: microdontia and macrodontia
- Tooth position alterations: impaction (excluding third molars), transposition, Displacement of maxillary canine (DMC), and ectopic eruption
- Tooth shape alterations: taurodontism, fusion, germination, dens invaginatus, dens evaginatus, short root anomaly, conoid lateral.

**Table 1:** Descriptive analysis

	Age	N	Gender	
			M	F
G1	8–10 years	138	64	74
G2	11–30 years	641	382	259

### Statistical analysis

The statistical analysis was performed using SPSS software package (Statistical Package for Social Sciences, version 16.0, SPSS Inc., Chicago, USA).

Descriptive statistics were used to describe both sample groups (G1 and G2) in terms of age, sex, and prevalence rate of dental anomalies; while for the comparative analysis, Spearman correlation coefficient was used to evaluate the associations between the different dental anomalies. The level of *p* value < 0.05 was considered statistically significant. For different dental anomalies, both, the prevalence, and ways of association were studied. To evaluate the association between dental anomalies and gender, the Chi-square test was used for the significative findings of this association.

## Results

No systematic error was found between the repeated digital measurements. The systematic error was reduced by the great experience of the examiner who was previously trained.

The sample size has been determined considering a suspected prevalence of 0.40 and a confidence level of 95. A minimum of 576 subject was needed for the sample size. A total of 779 patients were included, 459 females and 321 males (mean age of 15.1 ± 5.5 years). A total of 190 subjects (24.4%) had at least one dental anomaly, 115 females (60.5%) and 75 males (39.5%). Only one dental anomaly was noticed among 149 subjects (19.1% of the sample), 92 females (61.7%), and 57 males (38.3%). Two different anomalies were detected in 36 subjects (4.6% of the sample), while in only 5 subjects more than two different anomalies were observed (0.6%).

No statistically significant correlation was found between the occurrence of dental anomalies and gender (*p* > 0.05), as shown in Table 2.

**Table 2:** Prevalence rate of different anomalies in the whole sample (DA = dental anomaly)

Dental anomalies	Total sample			
	Prevalence N.—%			
	F (%)	M (%)	TOT (%)	%
No DA	344|59	245|41	589	75.6
DA	115|60.5	75|39.5	190	24.4
One DA	92|61.7	57|38.3	149	19.1
Two DA	20|55.6	16|44.4	36	4.6
More than two DA	3|60	2|40	5	0.6

Correlation between DA and gender: *p* = 0.241

The G1 presented at least one anomaly in 28 subjects (3.6%), while the G2 presented at least one dental anomaly in 162 subjects (20.7%). Both groups (Table 3) presented more frequently only one dental anomaly with no correlation between the gender (*p* > 0.05). The distribution of the observed dental anomalies is presented in Table 4. The most frequent anomalies in the whole sample were the following: hypodontia (69 subjects, 8.9%), dental impaction (59 subjects, 7.6%) and ectopic eruption (41 subjects, 5.3%). Among the findings, just the conoid laterals were identified as shape anomalies in 11 subjects (1.4%). No statistical difference was analyzed between the gender (*p* > 0.05).

**Table 3:** The prevalence rate of different anomalies in the two sub-groups

Dental anomalies	8–10 years old			> 10 years old		
	Prevalence N.%			Prevalence N.%		
	F (%)	M (%)	TOT (%)	F (%)	M (%)	TOT (%)
No DA	58|53	52|47	110|14.2	287|60	192|40	479|61.5
DA	16|57.1	12|42.9	28|3.6	95|58.6	67|41.4	162|20.7
One DA	13|56.5	10|43.5	23|2.9	79|62.7	47|37.3	126|16.1
Two DA	3|60.0	2|40.0	5|0.6	14|45.2	17|54.8	31|3.9
More than two DA	–	–	–	2|40.0	3|60.0	5|0.6

*DA* Dental anomaly

**Table 4:** Prevalence rate of different anomalies in the sample and in two sub-groups

Anomalies	Whole sample			8–10 years old			> 10 years old		
	F (%)	M (%)	TOT (%)	F (%)	M (%)	TOT (%)	F (%)	M (%)	TOT (%)
	NR|%	NR|%							
*Eruption and exfoliation anomalies*									
IT	40|67.8	19|32.2	59|7.6	4|66.7	2|33.3	6|3.6	36|67.9	17|32.1	53|8.6
DMC	6|60	4|40	10|1.2	1|50	1|50	2|1.4	5|62.5	3|37.5	8|1.2
TT	3|75	1|25	4|0.5	1|100	0|0	1|0.6	2|66.7	1|33.3	3|0.4
ETE	17|41.5	24|58.5	41|5.3	5|83.3	1|16.7	6|3.6	12|34.3	23|65.7	35|5.7
*Number anomalies*									
H	43|62.3	26|37.7	69|8.9	4|40	6|60	10|6	39|66.1	20|33.9	59|9.6
ST	4|45.5	5|54.5	9|1.2	0|0	2|100	2|1.2	4|57.1	3|42.9	7|1.1
O	1|25	3|75	4|0.5	0|	0|	0|0	1|25	3|75	4|0.6
*Shape anomalies*									
MAC	0|0	1|100	1|0.1	0|0	0|0	0|0	0|0	1|100	1|0.1
MIC	7|77.8	2|22.2	9|1.2	1|100	0|0	1|0.6	6|75	2|25	8|1.2
T	1|50	1|50	2|0.3	0|0	0|0	0|0	1|50	1|50	2|0.3
G	0|0	1|100	1|0.1	0|0	0|0	0|0	0|0	1|100	1|0.1
F	0|0	1|0	1|0	0|0	0|0	0|0	0|0	0|0	1|0.1
DI	2|100	0|0	2|0.3	0|0	0|0	0|0	2|100	0|0	2|0.3
DE	0|0	0|0	0|0	0|0	0|0	0|0	0|0	0|0	0|0
RA	3|60	2|40	5|0.6	1|100	0|0	1|0.6	2|50	2|50	4|0.6
CL	7|63.6	4|36.4	11|1.4	2|100	0|0	2|1.2	5|55.6	4|44.4	9|1.4

*IT* Impacted tooth, *DMC* Displacement of the maxillary canines, *TT* Tooth transposition, *ETE* Ectopic tooth eruption, *H* Hypodontia, *St* Sopranumerarum teeth, *O* Oligodontia, *MAC* Macrodontia, *MIC* Microdontia *T* Taurodontism *G* Germination *F* Fusion *DI* Dens Invaginatus, *DE* Dens Evaginatus, *RA* Root anomalie, *CL* Conoid Lateral

**Fig. 1:**
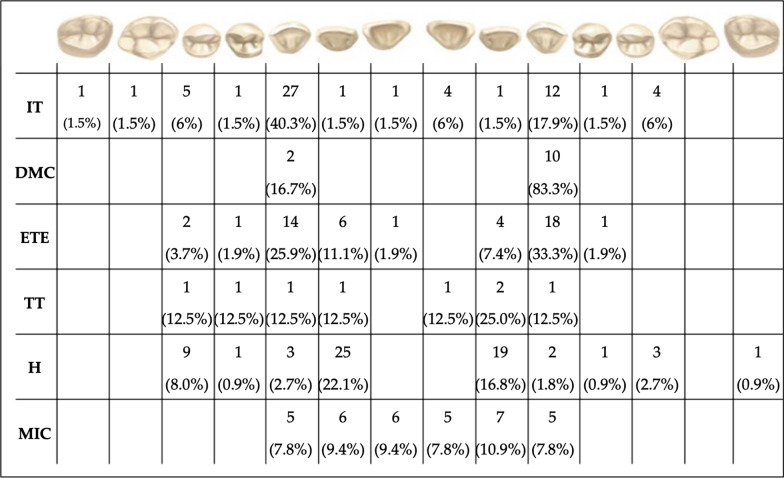
Number and prevalence of affected tooth for the most frequent anomalies found in the maxillary arch. *IT* Impacted tooth, *DMC* Displacement of the maxillary canines, *ETE* Ectopic tooth eruption, *TT* Tooth transposition, *H* Hypodontia, *MIC* Microdontia

**Fig. 2:**
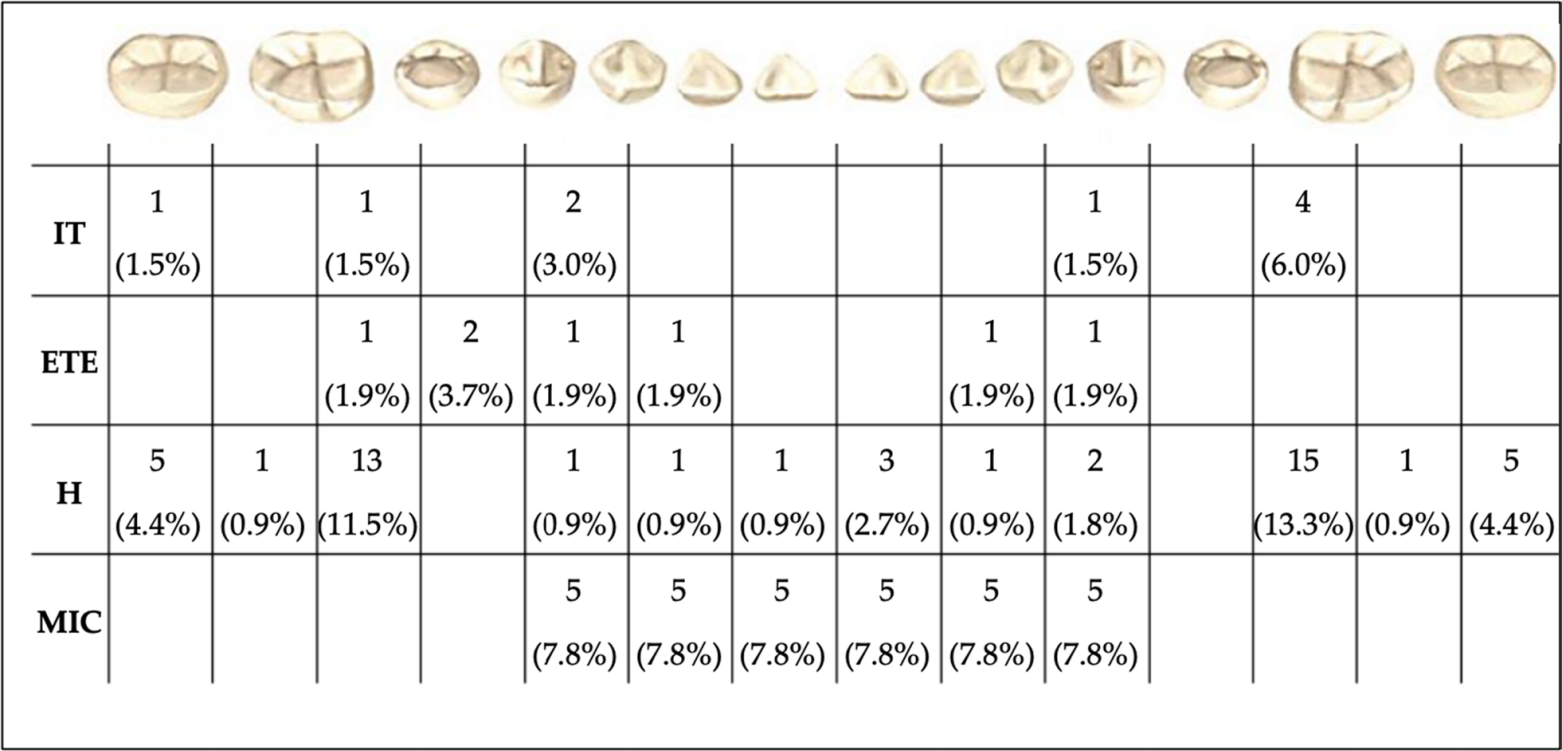
Number and prevalence of affected tooth for the most frequent anomalies found in mandibular arch. *IT* Impacted tooth, *ETE* Ectopic tooth eruption, *H* Hypodontia, *MIC* Mircrodontia

Figures 1 and 2 show the distribution of the different anomalies in the maxillary and mandibular arches. A total of 68 dental inclusions were found in the whole sample and the upper canines were the most frequently included teeth (n = 39; 57.3%). We found only one included canine in 28 subjects (3.5% of the whole sample) and bilateral canine inclusion in 6 subjects (0.7% of the whole sample) as shown in Fig. 1**.** The second most frequent included tooth was the upper second premolar (n = 9, 13.2%). 12 maxillary upper displacement canines were observed and the most common displacement one was the left maxillary canine (n = 10; 83.3%). The ectopic eruption was noticed in 54 dental elements. Upper canines were the most frequently ectopic teeth. (n = 32; 59.2%), in particular, 14 right upper canines (43.7%) and 18 left upper canines (56.3%). Only 12 subjects (54% of the sample) were characterized with only one ectopic canine and 10 subjects (46%) with both ectopic maxillary canines. The upper left canine was more frequent in the category of maxillary displacement canine (n = 10; 83%) respectively of the right maxillary canine (n = 2; 17%). 113 teeth were observed as agenesic in 69 subjects. Maxillary lateral incisors were the most frequently missing teeth, we found 44 missing laterals (38.9% of the missing teeth of the whole sample), the second most frequently missing teeth were the lower second premolars (n = 28; 24.7%) followed by upper second premolars (n = 12; 10%). In 12 subjects (42% of the sample) one missing lateral incisor was detected and in 16 patients (58%) the missing incisors were bilateral. Only 6 subjects presented one missing second lower premolar and 11 subjects were affected by bilateral missing second lower premolars, (Table 4). The correlation between the most common dental anomalies is shown in Table 5.

**Table 5:** Association between dental anomalies

	DMC	H	MIC	TT	ETE	CML
*Spear man’s rho*						
IT						
Correlation coefficient	− .033	.047	.014	− .021	− .002	.048
Sig. (2-tailed)	.363	.187	.687	.567	.949	.181
N	779	779	779	779	779	779
DMC						
Correlation coefficient		.005	− .012	− .008	.024	− .014
Sig. (2-tailed)		.898	.731	.819	.500	.704
N		779	779	779	779	779
H						
Correlation coefficient			.135**	− .022	.048	.116**
Sig. (2-tailed)			.000	.533	.182	.001
N			779	779	779	779
MIC						
Correlation coefficient				− .008	.028	.191**
Sig. (2-tailed)				.829	.430	.000
N				779	779	779
TT						
Correlation coefficient					0.64	− .009
Sig. (2-tailed)					.077	.811
N					779	779
ETE						
Correlation coefficient						.069
Sig. (2-tailed)						.053
N						779

*IT* Impacted tooth, *DMC* Displacment of the maxillary canines, *ETE* Ectopic tooth eruption, *TT* Tooth tranposition, *H* Hypodontia, *MIC* Mircrodontia

* the correlation is significant at .05, ** the correlation is significant at .01

There were significant association among different anomalies (sig. (2-tailed) < 0.01) like hypodontia and microdontia, hypodontia and conoid upper laterals, microdontia and conoid upper laterals.

## Discussion

The aim of the present investigation was to provide evidence about the prevalence and the distribution of different dental anomalies in the Albanian population of growing subjects and adults. Several studies evaluated the frequency of DA in orthodontic or paediatric subjects, the nature of the examined subjects influenced the prevalence rates of the examined anomalies, but it did not necessarily reflect the prevalence in the general population. Furthermore, there are no studies in Albania that examined the prevalence of dental anomalies. The panoramic radiography examination represents an easy method for identifying some of those anomalies, particularly those that cannot be observed during an intraoral examination, such as dental agenesis or dental inclusion. The results of this study demonstrated that 24.4% of evaluated patients presented at least one DA, revealing a considerable frequency in this population. This result is also in agreement with other studies which show a similar prevalence: Laganà et al. in an Italian population [7], Drenski Balija et al. in a Croatian population [12]. Other studies like Wagner et al. [13], Baron et al. [8], Shayan [14], Gupta et al. [15], reported a greater prevalence, these higher percentages could be related to a different methodology that the authors have carried out also including the photographic images which have also highlighted possible structural anomalies such as MIH, fluorosis or the inclusion of root dilacerations, DA not included in this research. Other studies reported lower percentages probably due to racial differences but also to consideration of fewer anomalies when examining [5, 9, 16]. The terms for congenital absence of teeth listed in order of increasing severity are hypodontia, oligodontia, and anodontia [5]. Dental agenesis was considered in the absence of the permanent tooth or the dental germ, taking into consideration the age of the patient. Qligodontia was observed in only four patients and no case of anodontia was found. Hypodontia was the most frequent dental anomaly in our sample (n = 59; 9.6%). When comparing this study with others performed in Europe, these findings are similar to studies carried out in France, Slovenia and Croatia [8, 9, 12]. The amount of missing teeth reported varies according to ethnicity [17]. In a systematic review, Khalaf Krecent et al. determined that 6.4% of people worldwide have hypodontia. Africa had the highest prevalence of hypodontia (13.4%), followed by Europe (7.0%), Asia (6.4%), and Australia (6.3%). North America (5.0%)) and Latin America and the Caribbean (4.4%). Mandibular second premolars, maxillary lateral incisors, and maxillary second premolars were the most frequently impacted teeth [18].

In this study, the exfoliation and eruption alteration was very common, in fact, dental inclusion was the second most frequent dental anomaly after hypodontia (59 subjects, 7.6%). The upper canines were the most affected teeth. Our data are similar to Wagner (6.4%) [13], Pallikaraki (5.7%) results [6]. In order to study the anomalies, especially the exfoliation and the eruption anomalies, we divided the sample in two subgroups: G1 with 138 subjects aged between 8 and 10 years old; G2 with 641 subjects aged between 11 and 30 years old. The displacement of the canine in this study was found in 12 subjects: 1.3% of the whole sample and 8.6% of the early age group. The displacement of the canine represents the first phase of inclusion and a moment where it is possible to intervene to enhance a spontaneous eruption. Dental impaction may be avoided by using radiographic clues for early canine dis-placement diagnosis and treatment planning. [19].

Proffit [20] mentioned that the most common abnormality is variation in dental size, particularly of the maxillary lateral incisors. In our study, the prevalence of peg-shaped maxillary lateral incisors was 1.4%, making it the most frequent shape anomaly in our sample. This anomaly is often correlated with a palatal displaced canine. For this reason, patients who present this anomaly should be subjected to a more accurate investigation since there aren’t specific clinical signs.

Significant correlations between various dental anomalies were found in this study, suggesting that these disorders may share a common etiological cause. Marra et al. [21] found a significantly higher prevalence of microdontia of the maxillary lateral incisors (*p* < 0.001) and delayed tooth development (*p* = 0.0001) in subjects that present agenesis tooth respect subjects compared with a non-agenesis control group.

Other investigations have demonstrated a direct connection between the maxillary lateral incisors' agenesis and the maxillary canine's displacement [7, 22]. Early detection of dental anomalies is important because they can interfere with the normal process of tooth eruption increasing the risk of dental inclusions, malpositions and malocclusion.

In the present study, we provided a definition for each dental anomaly. However, the diagnosis of some AD, such as macrodontia or microdontia, dens invaginatus and dens evaginatus is dependent on the examiner's interpretation, which is a limitation of this study. Another important aspect is that the dental anomalies were mainly observed with panoramic radiographs. Most of the DA are clearly detectable by this type of radiological examination, however, some anomalies such as structural ones, require a more appropriate intraoral examination. For this reason, in this study, we did not include anomalies such as amelogenesis imperfect or imperfect dentinogenesis. Nevertheless, it is important to emphasise that the present work represents the first epidemiological study of dental anomalies in the Albanian population, and its strength is certainly the high sample size.

## Conclusions

Epidemiological studies may provide valuable information for the identification of the population affected by various anomalies and can also contribute to finding possible associations between different anomalies. The results of our study on dental anomalies in Albanian orthodontic patients suggest: 24.4% of patients had at least one examined anomaly and 4.6% of the sample had more than one anomaly. Hypodontia was the most frequent anomaly, followed by tooth impaction and ectopic eruption.

Early diagnosis of dental anomalies can contribute to prevent their complications and to establish the most suitable therapy to achieve functional occlusion. The clinician needs to be aware that other dental anomalies may coexist with hypodontia.

## Data Availability

The datasets used and/or analyzed during the current study are available from the corresponding author on reasonable request.
